# Supplementary material for: The correlation between cellular O-GlcNAcylation and sensitivity to O-GlcNAc inhibitor in colorectal cancer cells
Source: PLoS One. 2024 Oct 16;19(10):e0312173. doi: 10.1371/journal.pone.0312173 (PMC11482669; doi:10.1371/journal.pone.0312173)
Supplement: S1 Raw images — (PDF) [file pone.0312173.s001.pdf]

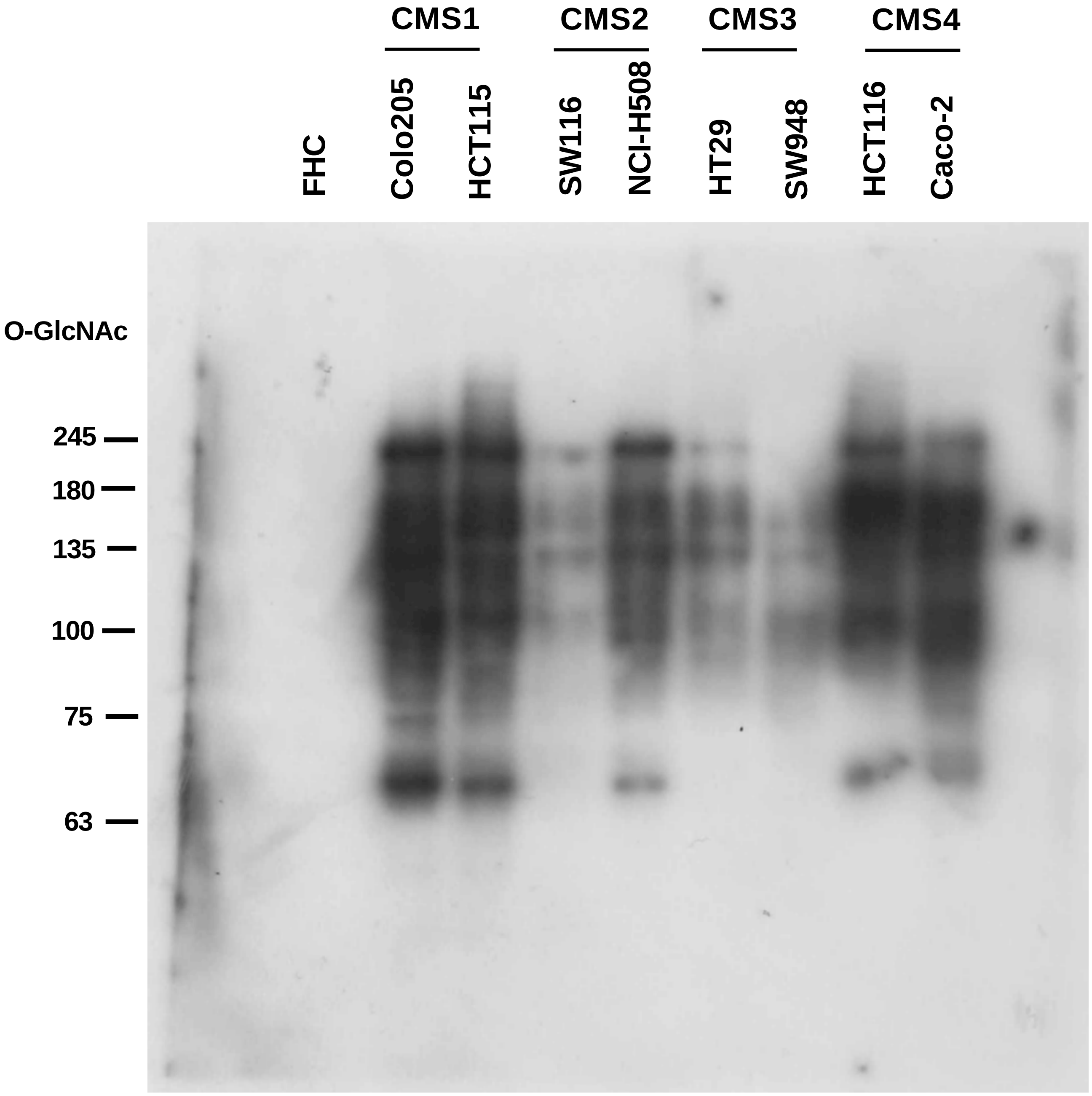

Shown in Fig 2 Panel O-GlcNAc and S1 Fig A Panel O-GlcNAc

This image was captured by using Ricoh MP2555 Scanner

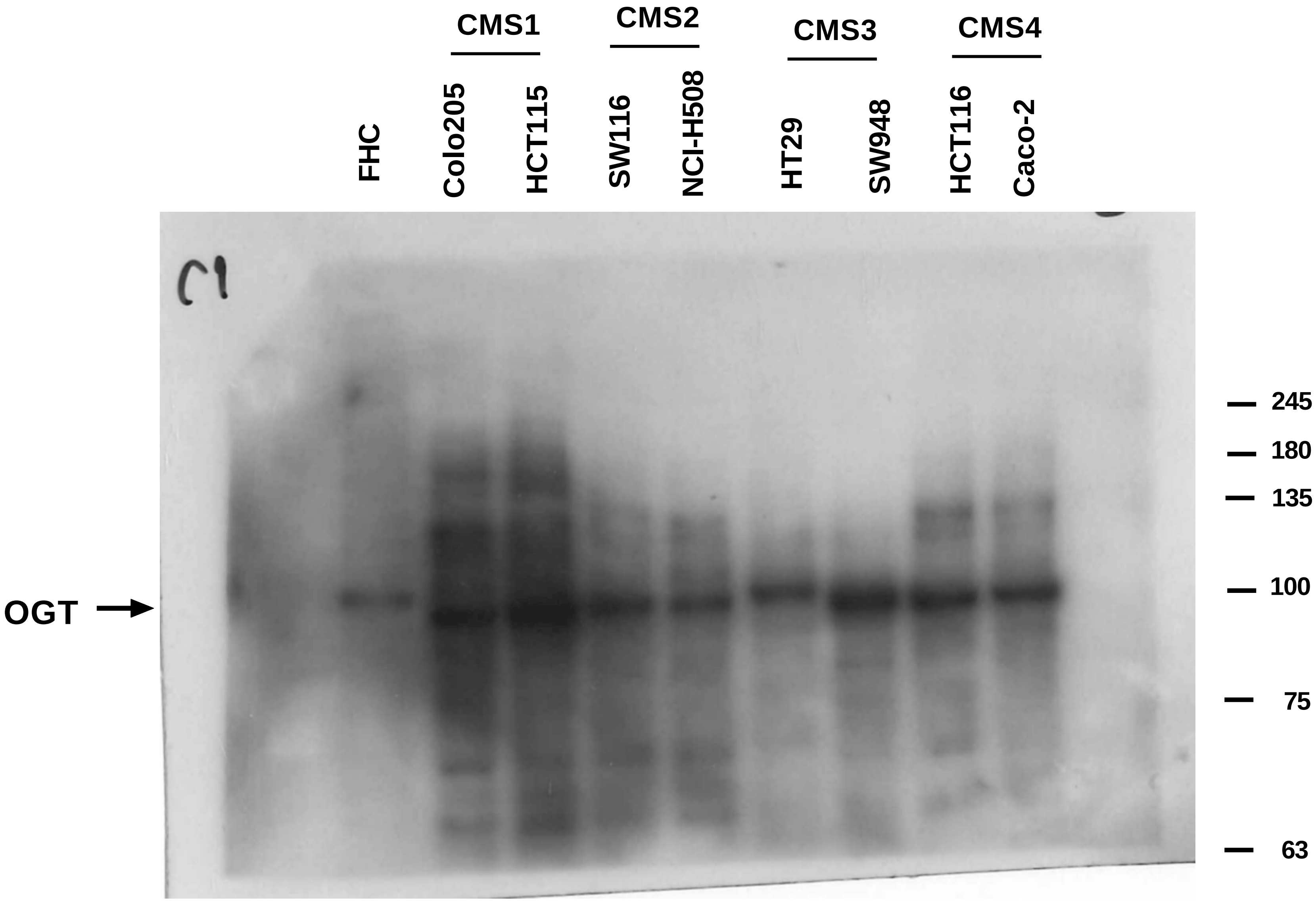

Shown in Fig 2 Panel OGT and S1 Fig A Panel OGT

This image was captured by using Ricoh MP2555 Scanner

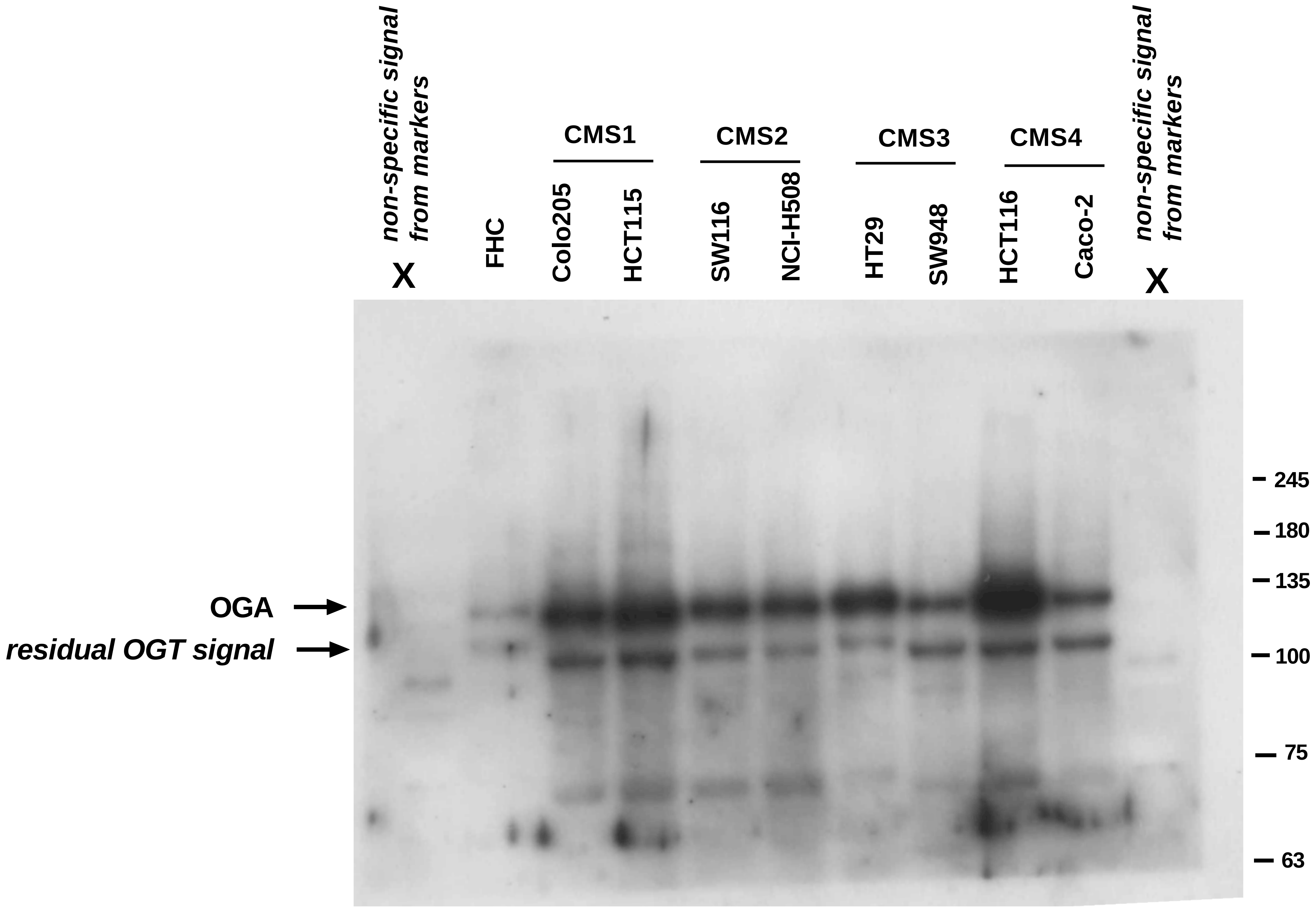

Shown in Fig 2 Panel OGA and S1 Fig A Panel OGA

This image was captured by using Ricoh MP2555 Scanner

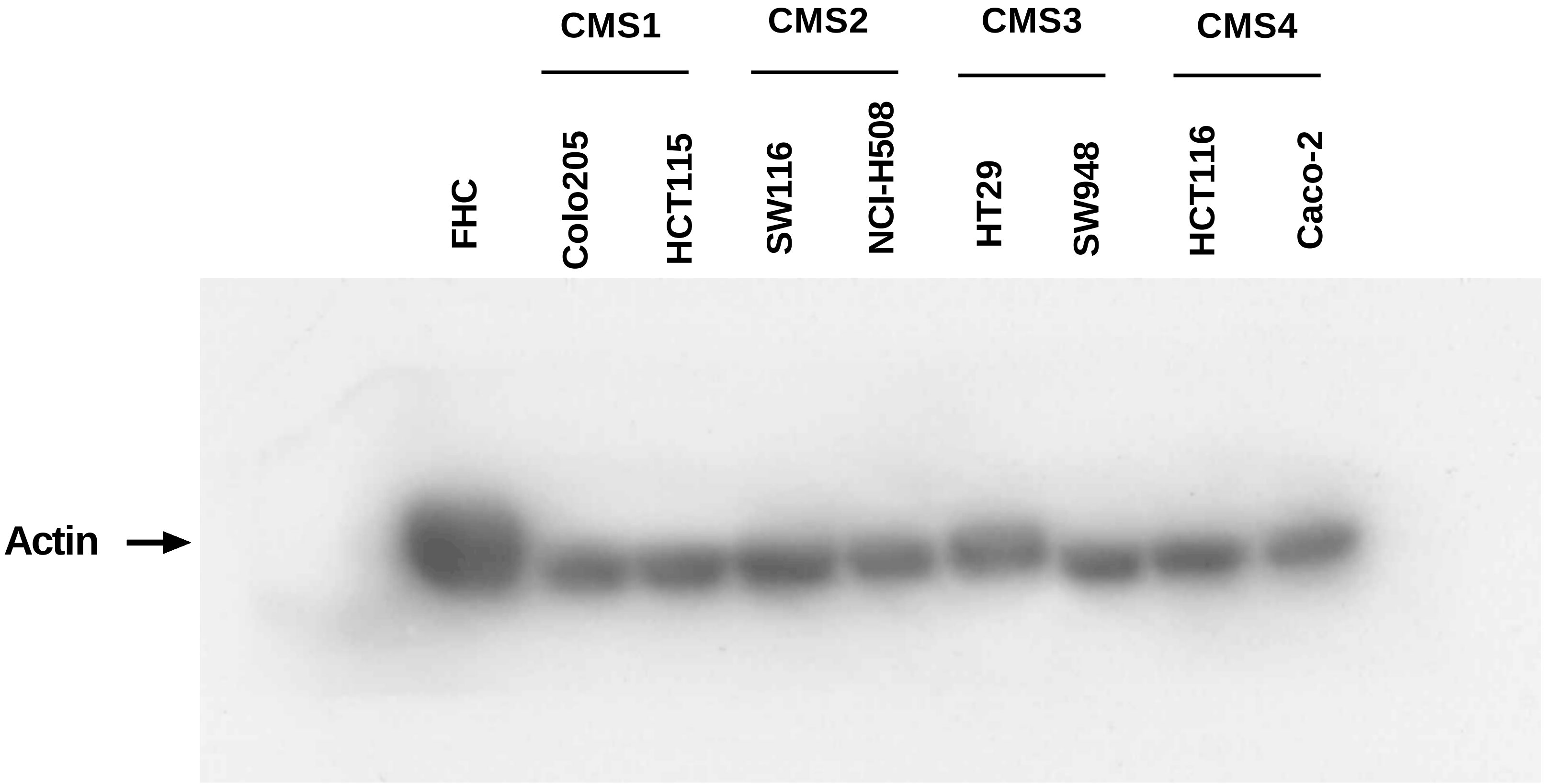

Shown in Fig 2 Panel Actin and S1 Fig A Panel Actin

This image was captured by using Ricoh MP2555 Scanner

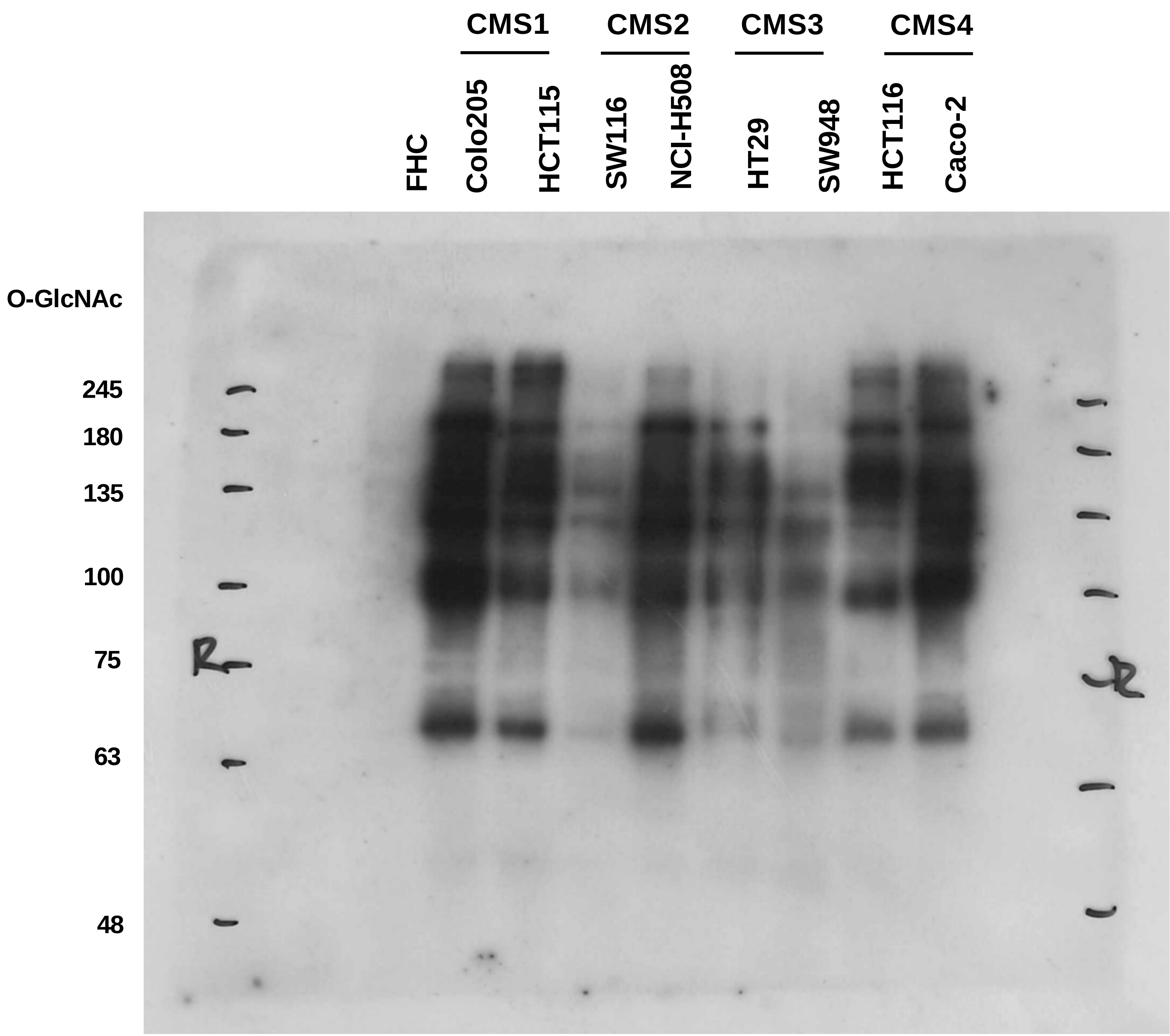

Shown in S1 Fig B Panel O-GlcNAc

This image was captured by using Ricoh MP2555 Scanner

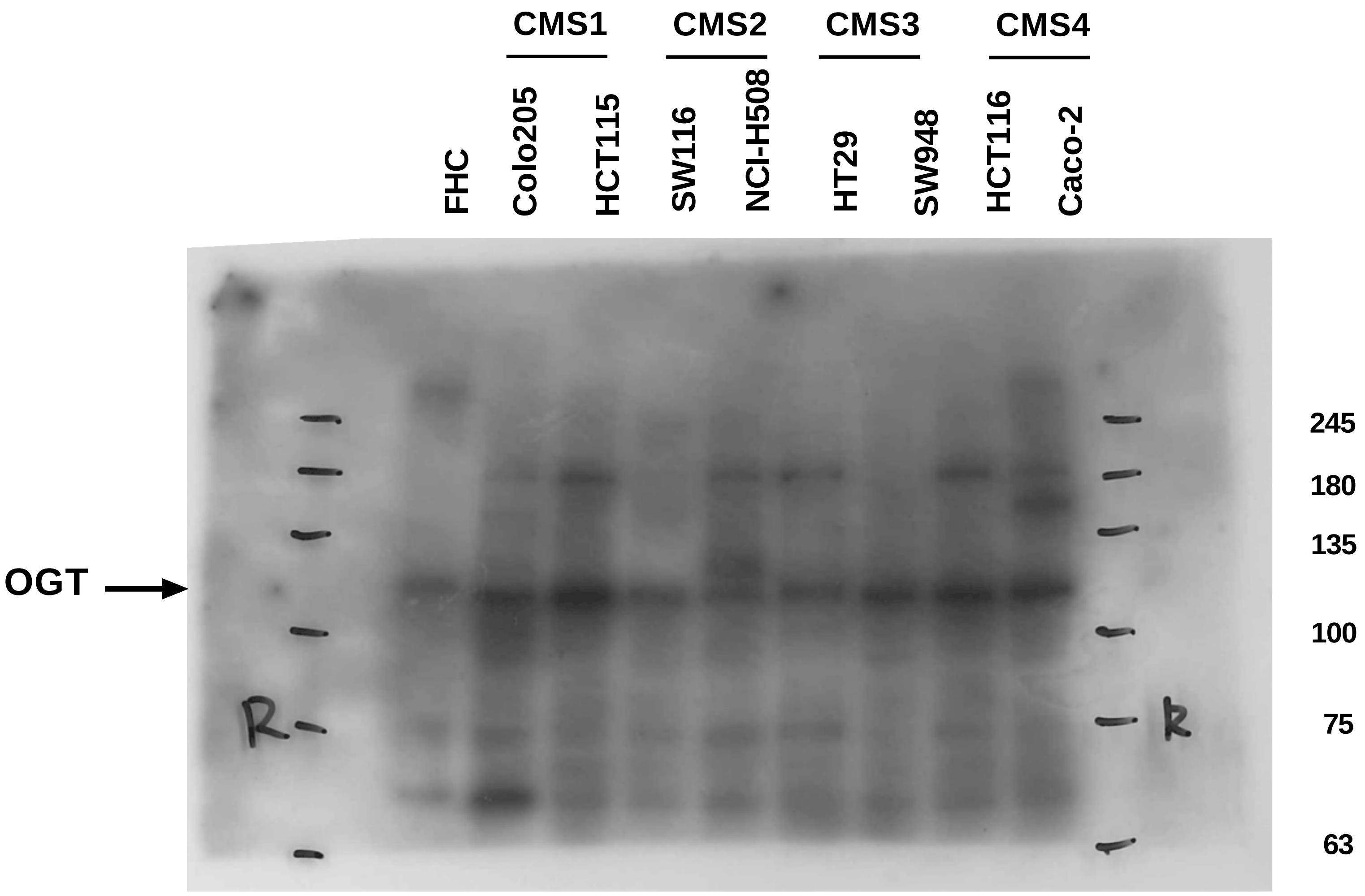

Shown in S1 Fig B Panel OGT

This image was captured by using Ricoh MP2555 Scanner

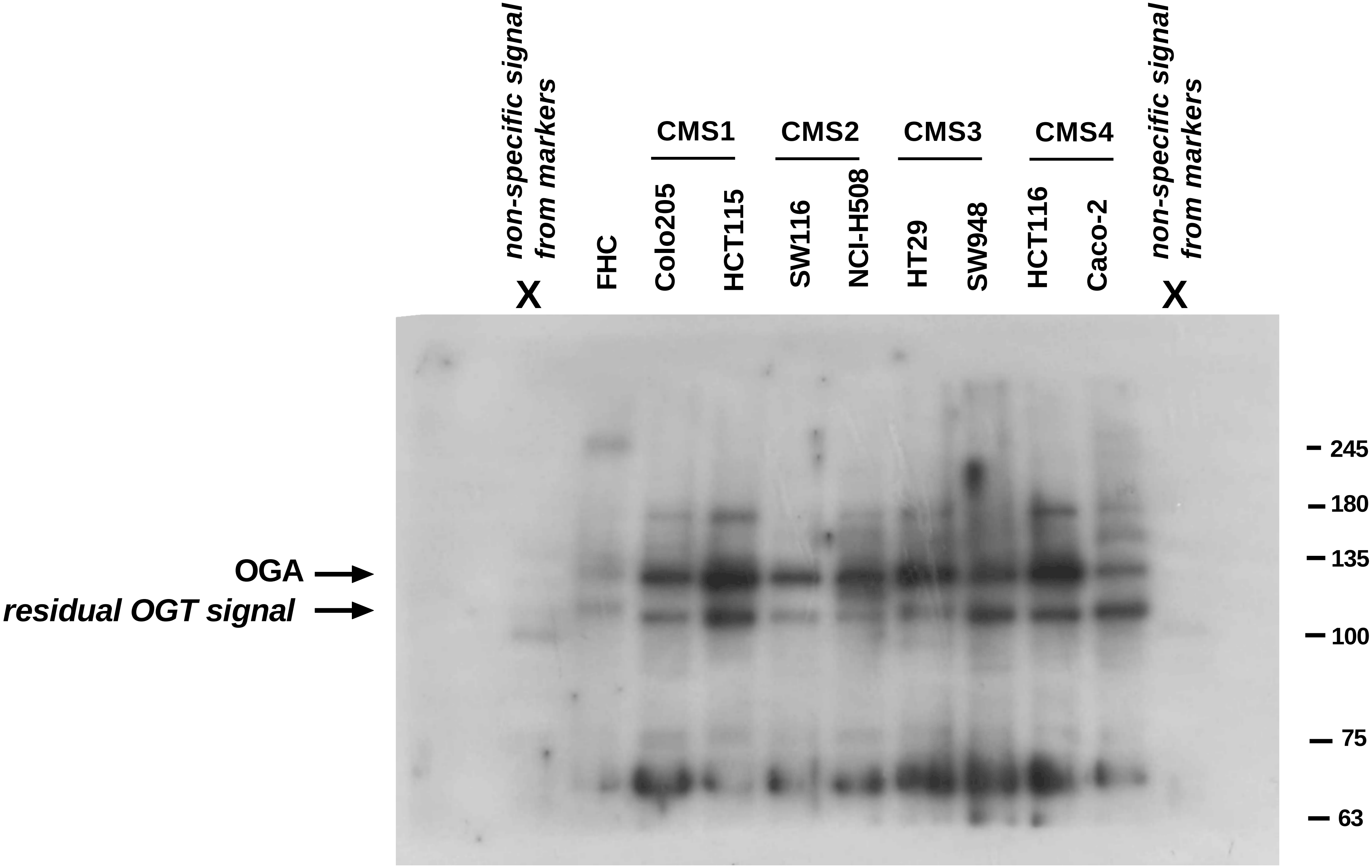

Shown in S1 Fig B Panel OGA

This image was captured by using Ricoh MP2555 Scanner

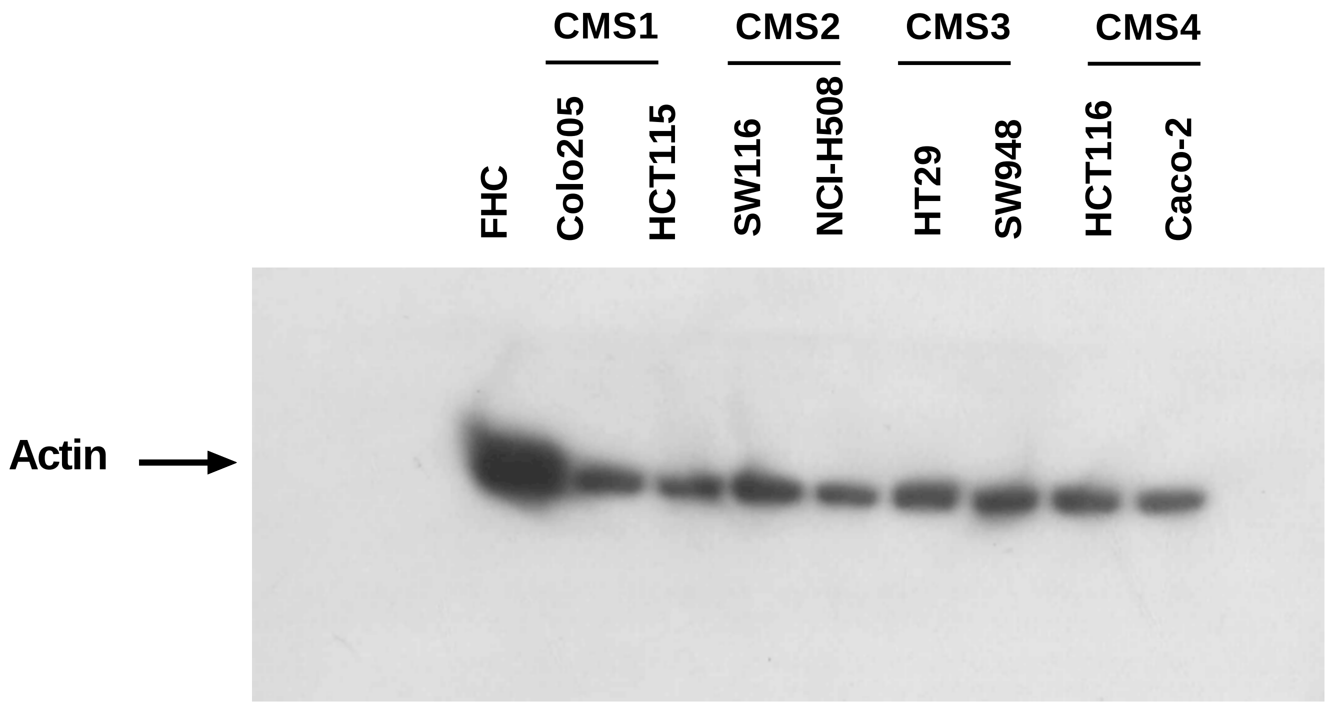

Shown in S1 Fig B Panel Actin

This image was captured by using Ricoh MP2555 Scanner

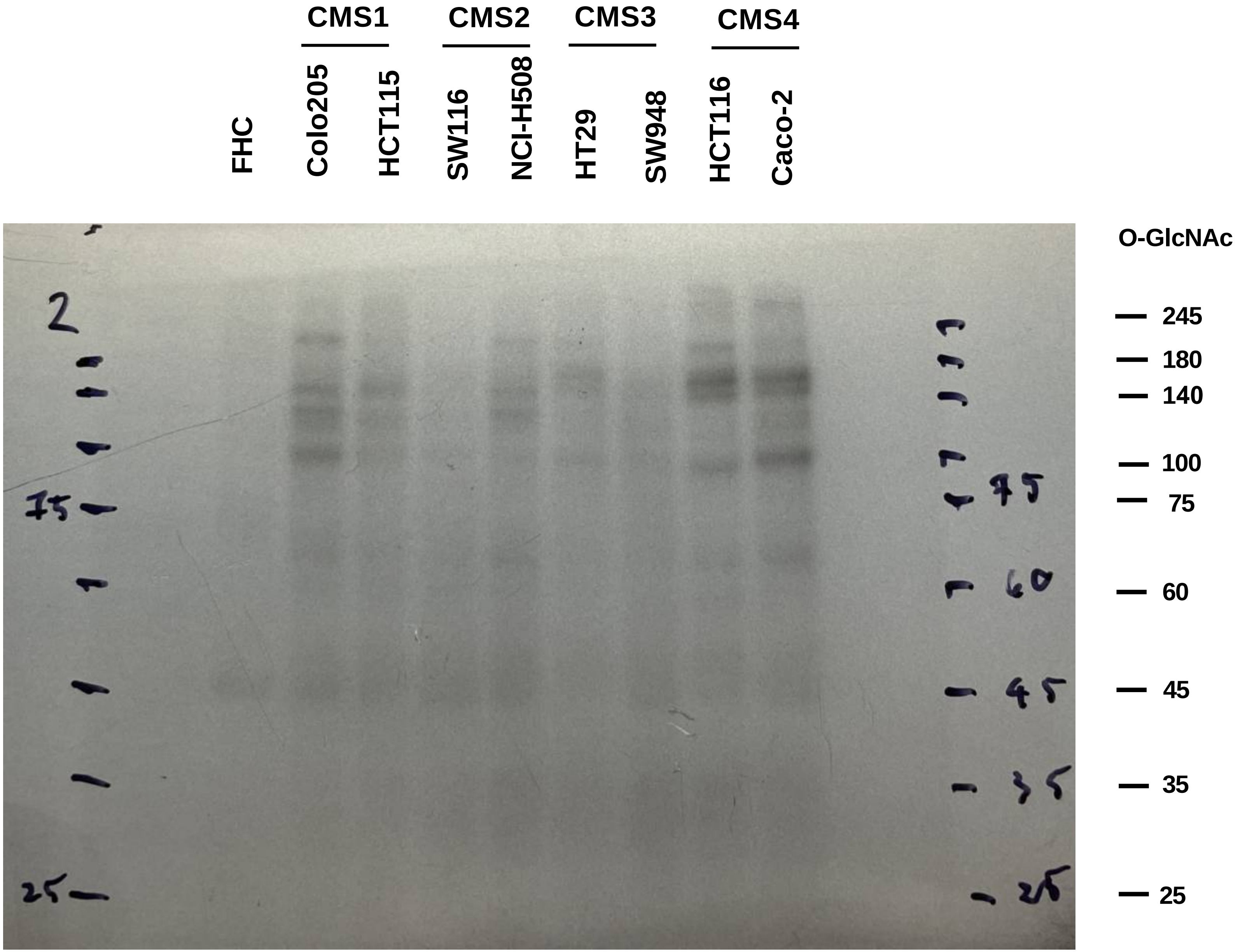

Shown in S1 Fig C Panel O-GlcNAc

The film was positioned on top of a bright surface (white light).
The image was subsequently captured using a digital camera (iPhone 12).

non-specific signal from markers

X

	CMS1		CMS2		CMS3		CMS4	
FHC	Colo205	HCT115	SW116	NCI-H508	HT29	SW948	HCT116	Caco-2

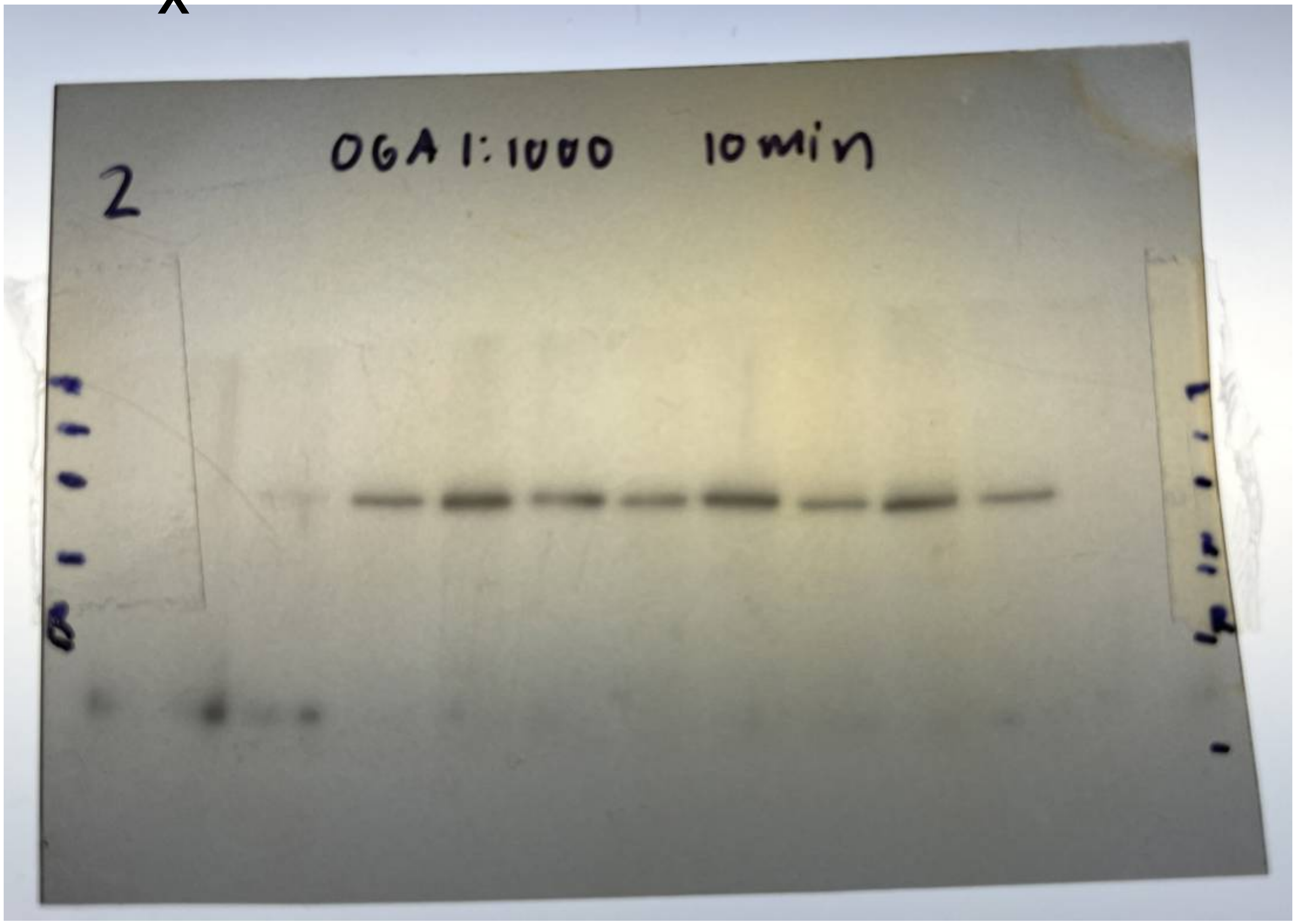

OGA →

245
180
140
100
75
60

Shown in S1 Fig C Panel OGA

The film was positioned on top of a bright surface (white light).
The image was subsequently captured using a digital camera (iPhone 12).

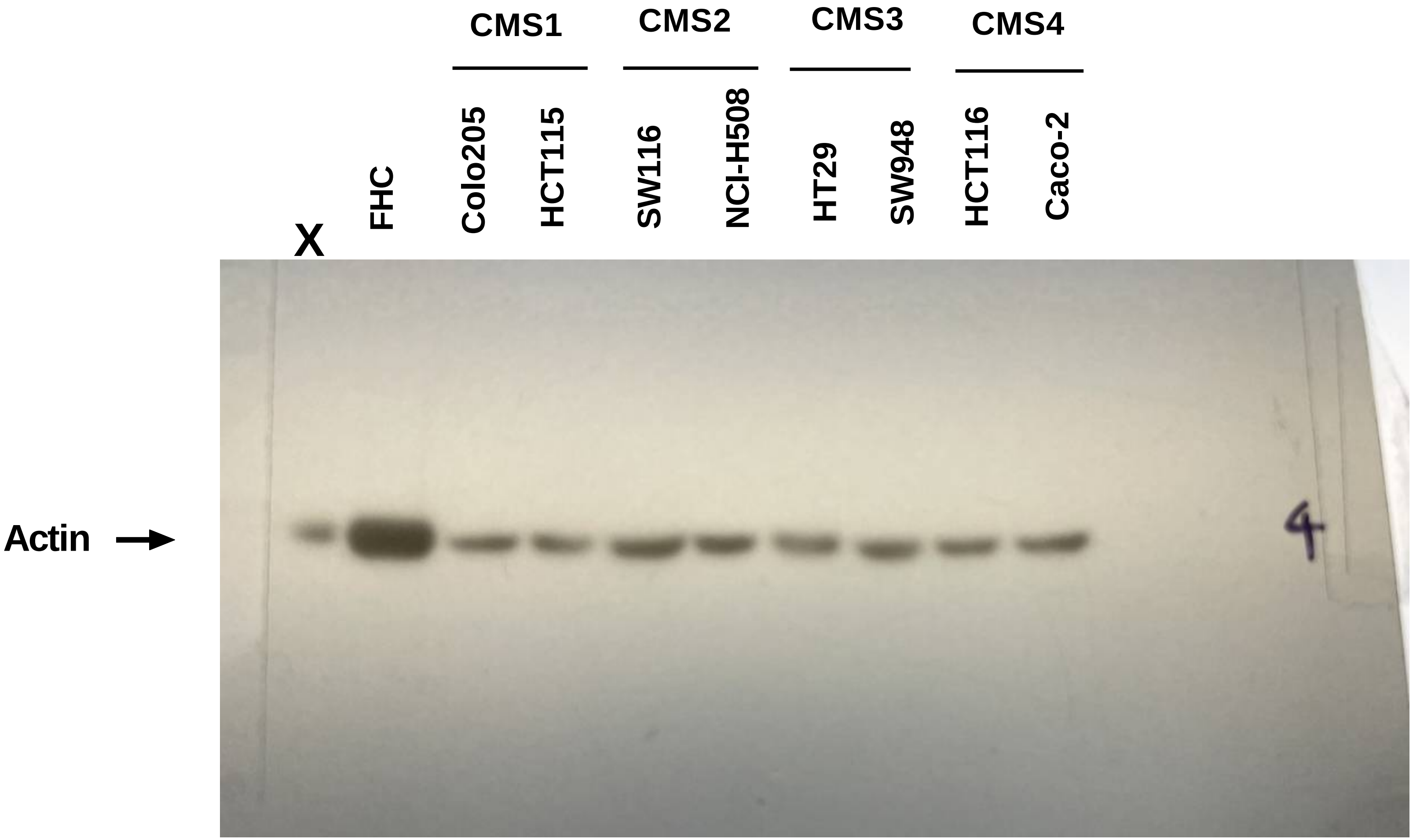

Shown in S1 Fig C Panel Actin (upper panel)

The film was positioned on top of a bright surface (white light).
The image was subsequently captured using a digital camera (iPhone 12).

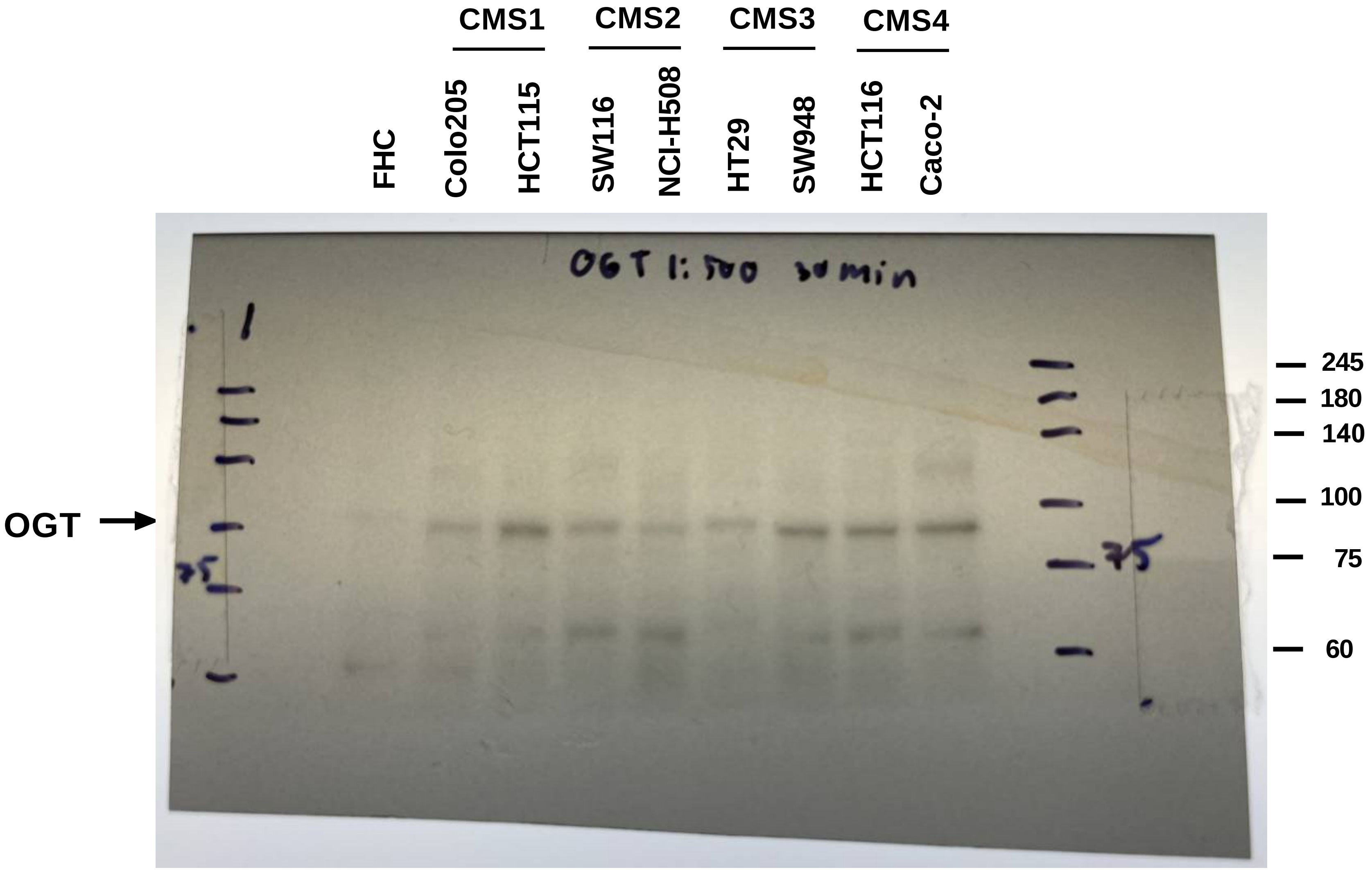

Shown in S1 Fig C Panel OGT

The film was positioned on top of a bright surface (white light).
The image was subsequently captured using a digital camera (iPhone 12).

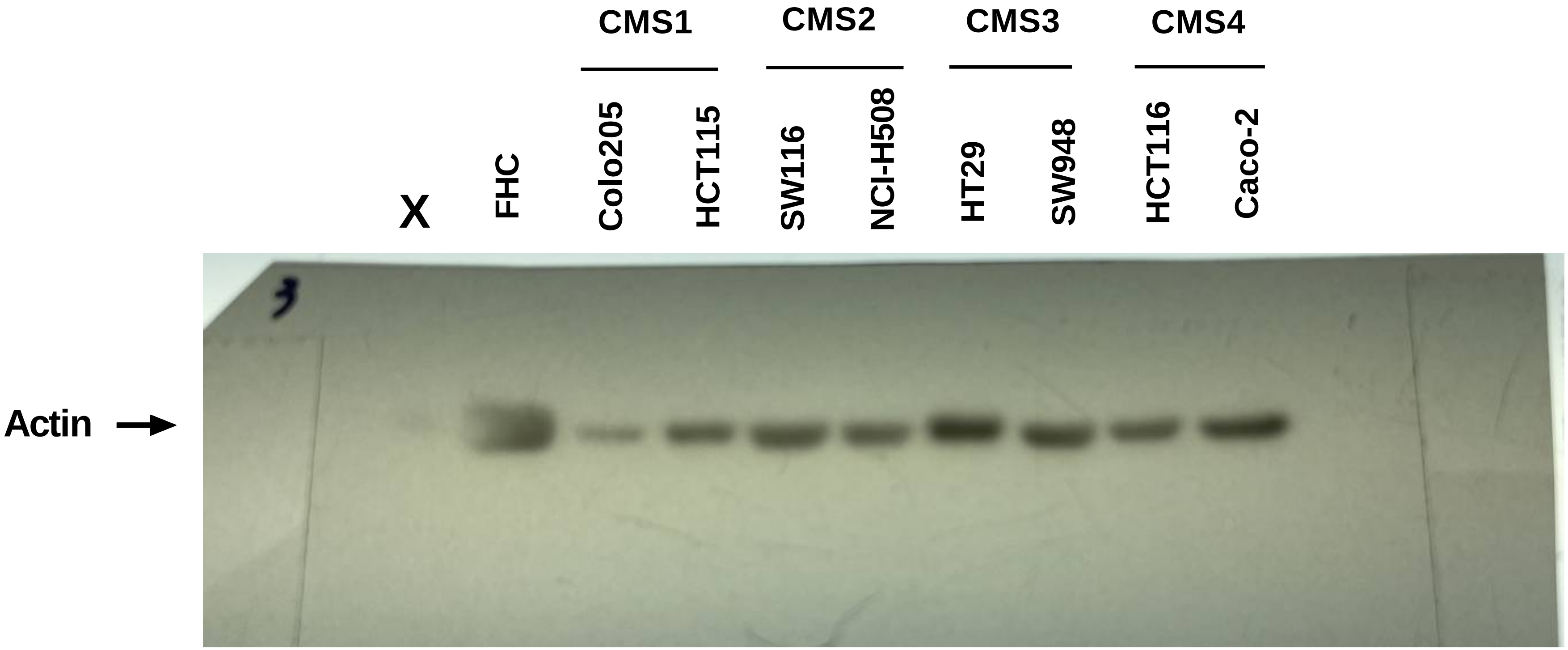

Shown in S1 Fig C Panel Actin (Lower panel)

The film was positioned on top of a bright surface (white light).
The image was subsequently captured using a digital camera (iPhone 12).

Lane:	1	2	3	4	5	6	7	8
Os(µM):	-	10	20	-	-	-	10	20
Re(µM):	-	-	-	1	2	10	1	2

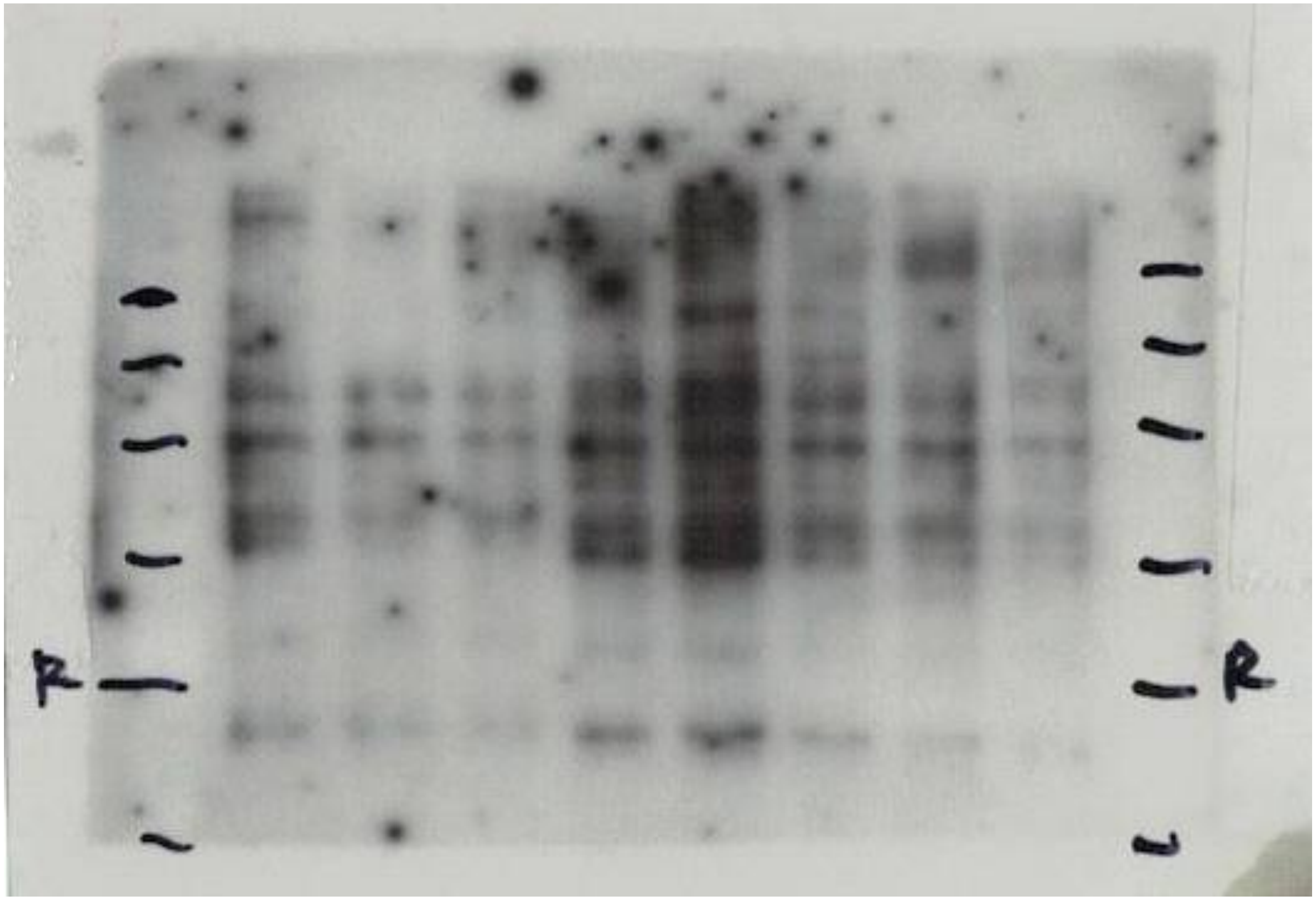

Shown in Fig 5C Panel O-GlcNAc

This image was captured by using Ricoh MP2555 Scanner

Lane:	1	2	3	4	5	6	7	8
Os(μM):	-	10	20	-	-	-	10	20
Re(μM):	-	-	-	1	2	10	1	2

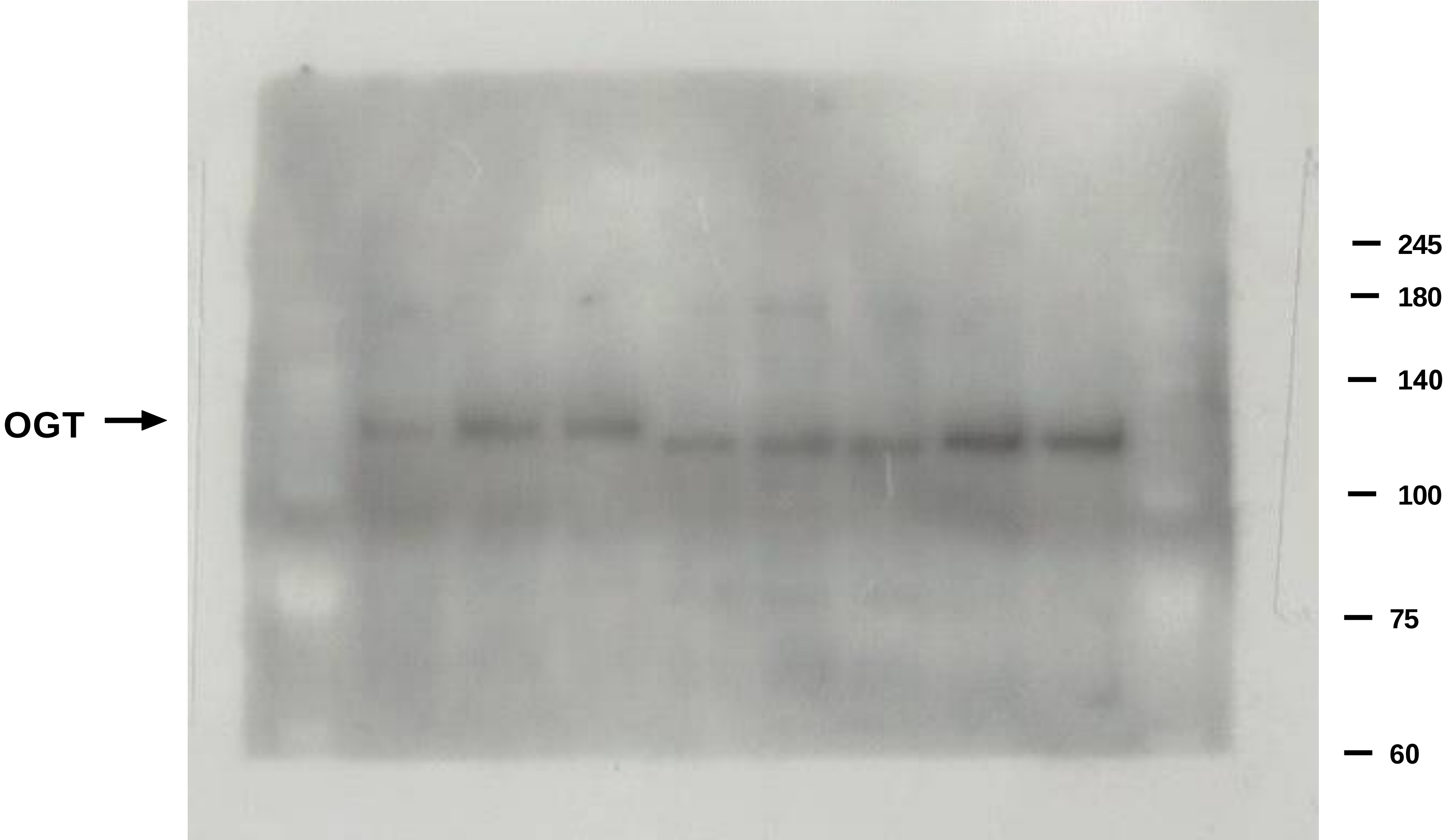

Shown in Fig 5C Panel OGT

This image was captured by using Ricoh MP2555 Scanner

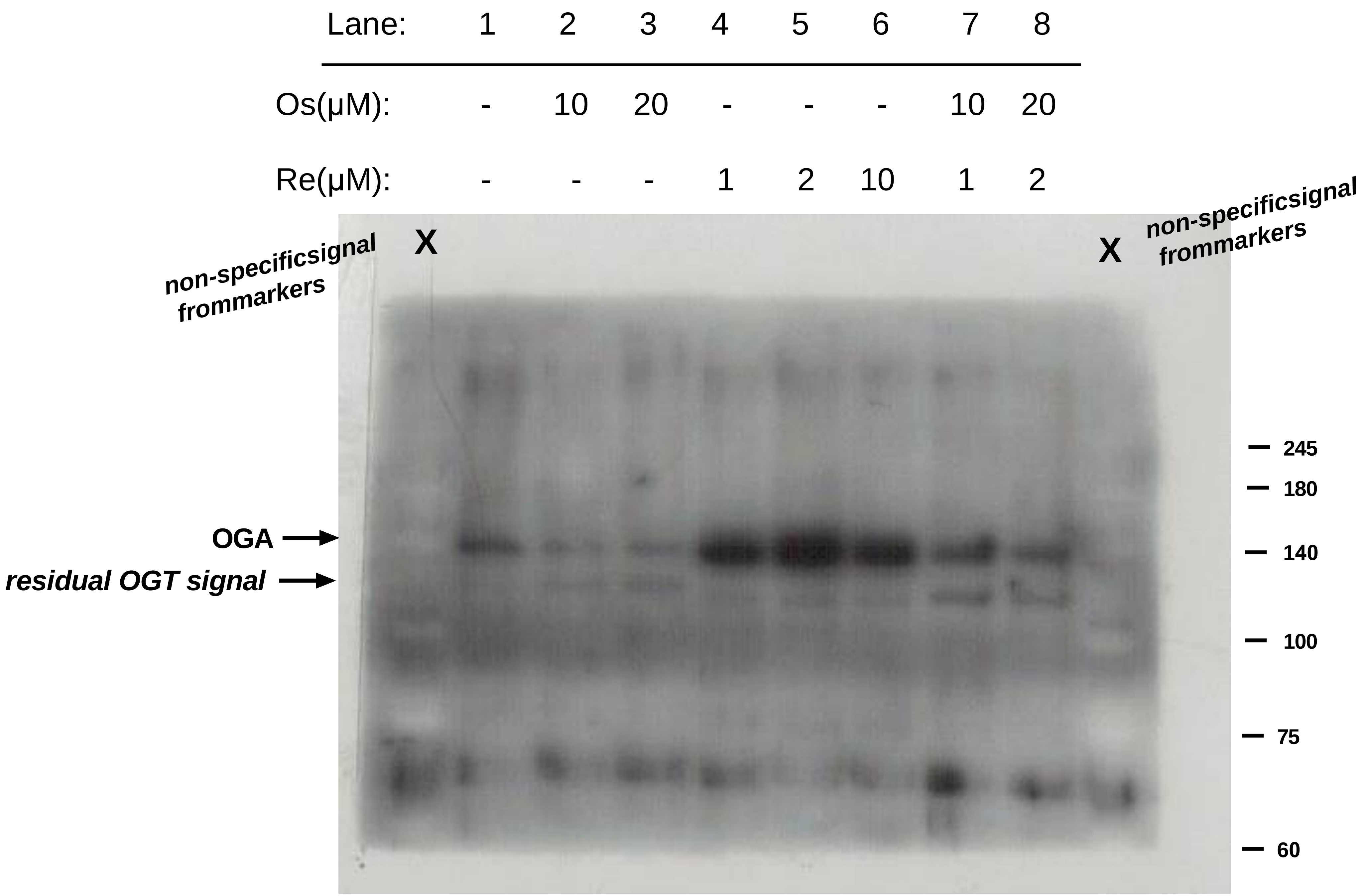

Shown in Fig 5C Panel OGA

This image was captured by using Ricoh MP2555 Scanner

Lane:	1	2	3	4	5	6	7	8
Os(μM):	-	10	20	-	-	-	10	20
Re(μM):	-	-	-	1	2	10	1	2

Actin →

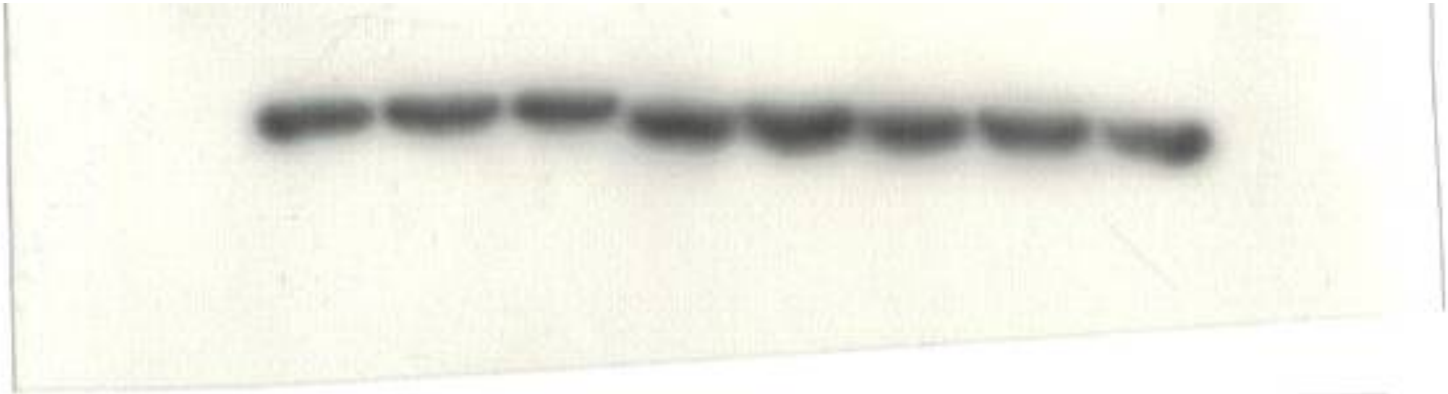

Shown in Fig 5C Panel Actin

This image was captured by using Ricoh MP2555 Scanner

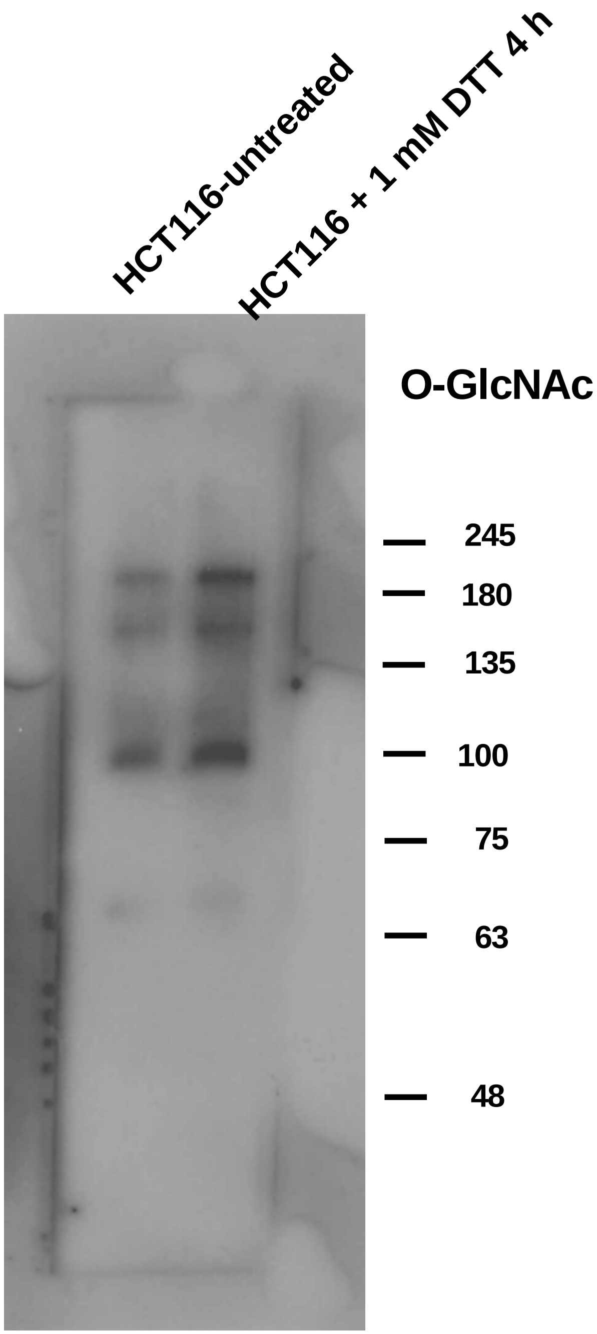

Shown in S2 Fig Lane 1-2 Panel O-GlcNAc

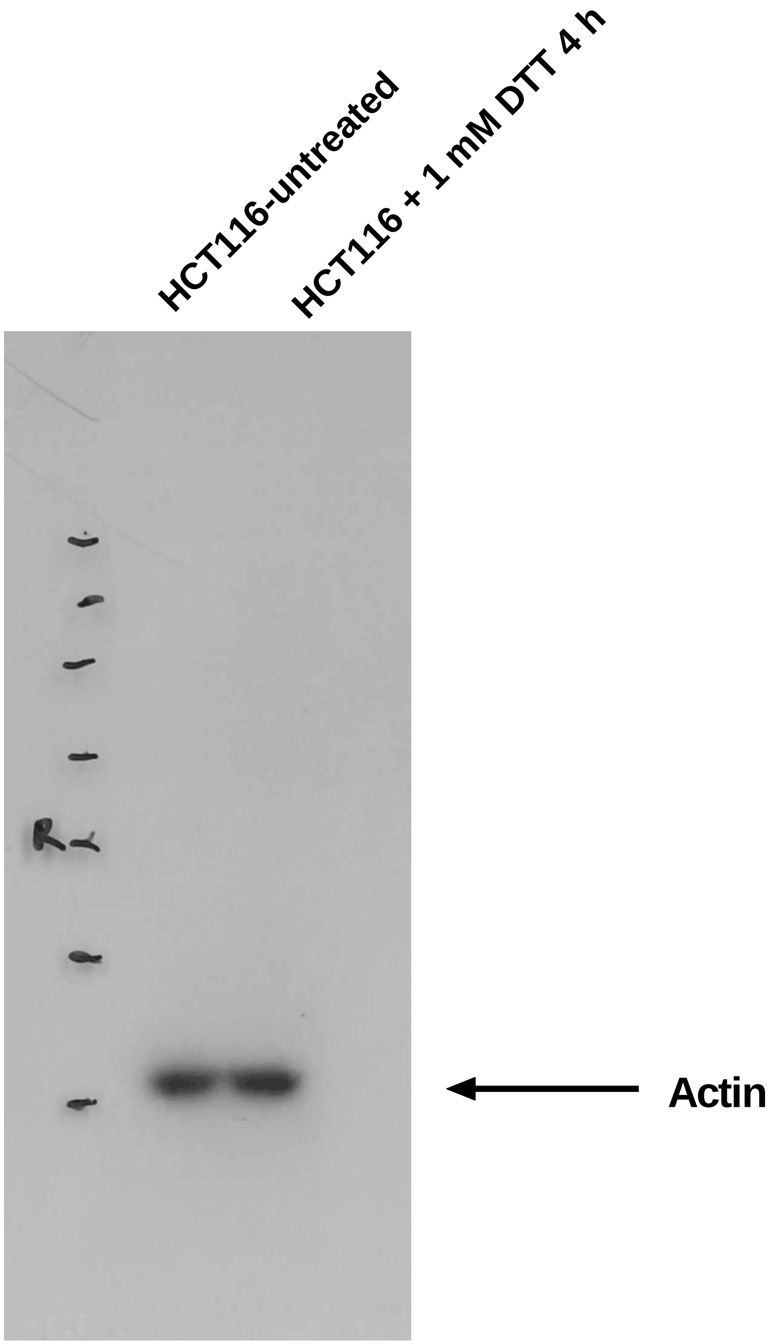

Shown in S2 Fig Lane 1-2 Panel Actin

This image was captured by using Ricoh MP2555 Scanner

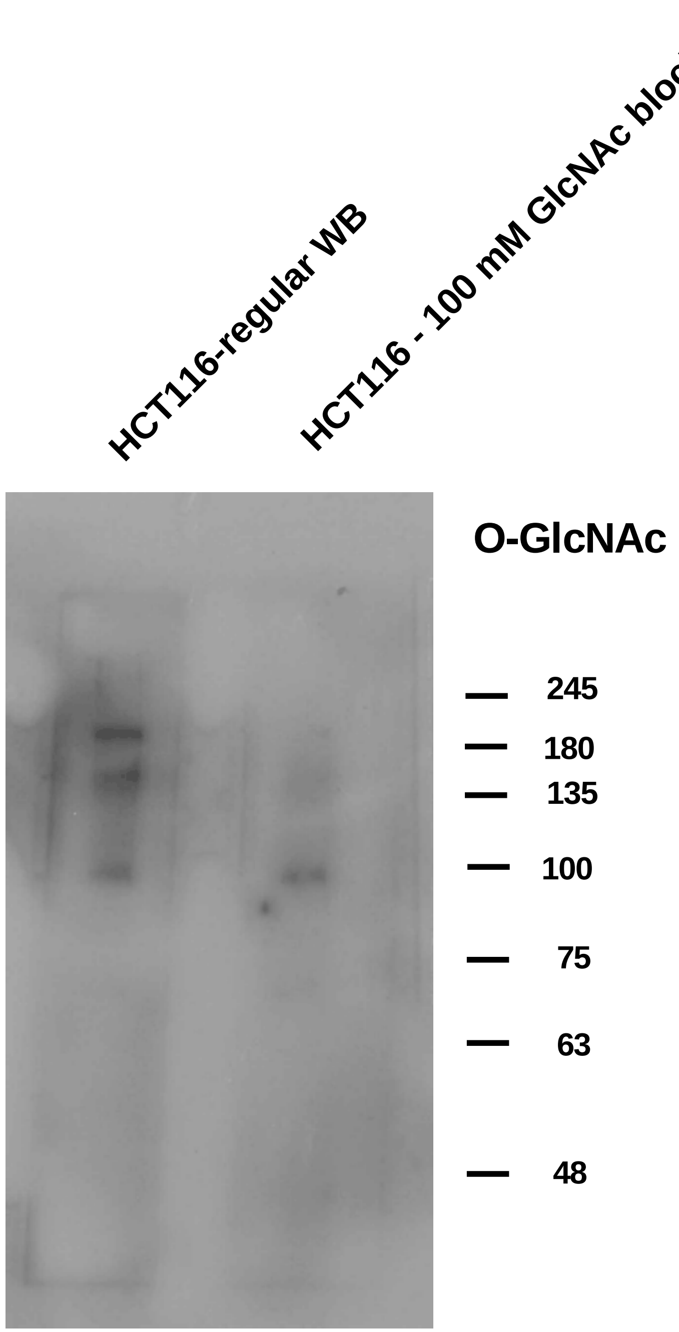

Shown in S2 Fig Lane 3-4 Panel O-GlcNAc

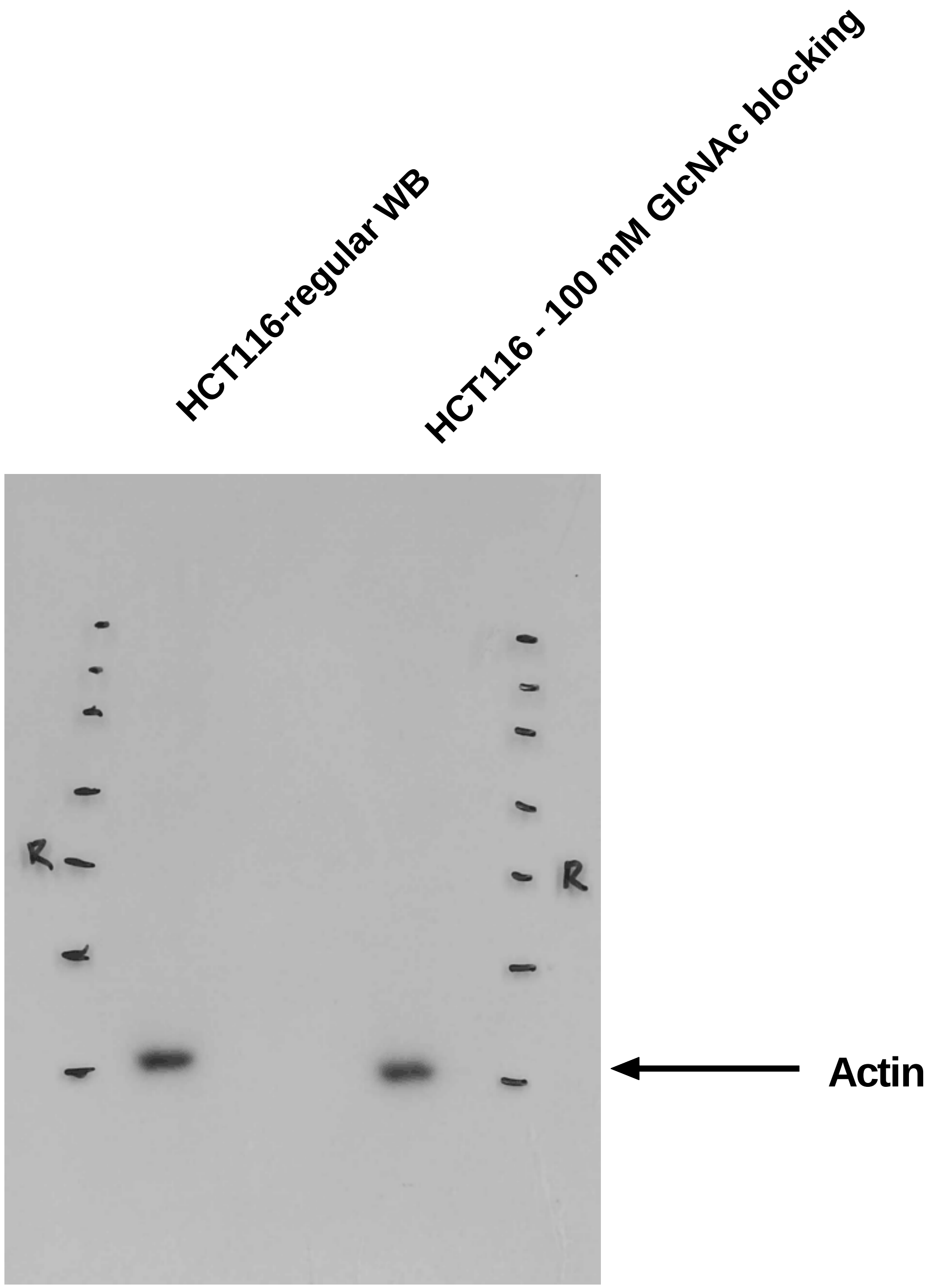

Shown in S2 Fig Lane 3-4 Panel Actin

This image was captured by using Ricoh MP2555 Scanner

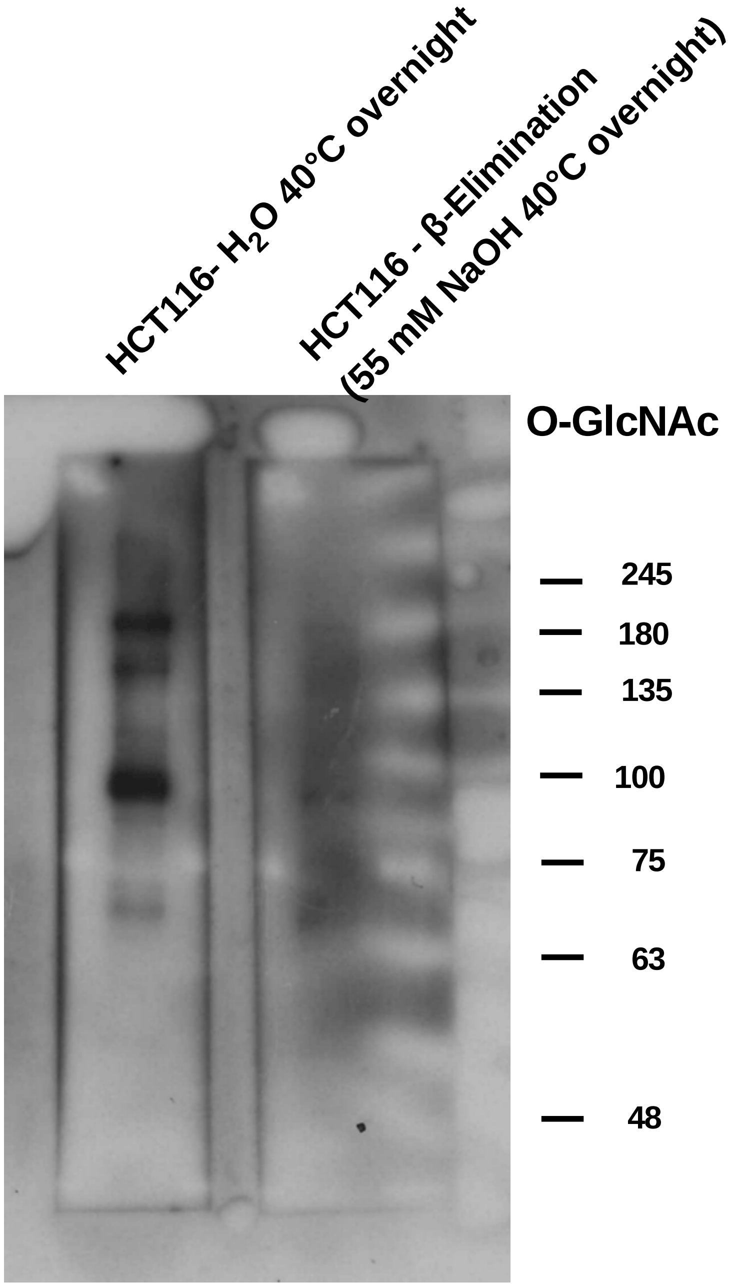

Shown in S2 Fig Lane 5-6 Panel O-GlcNAc

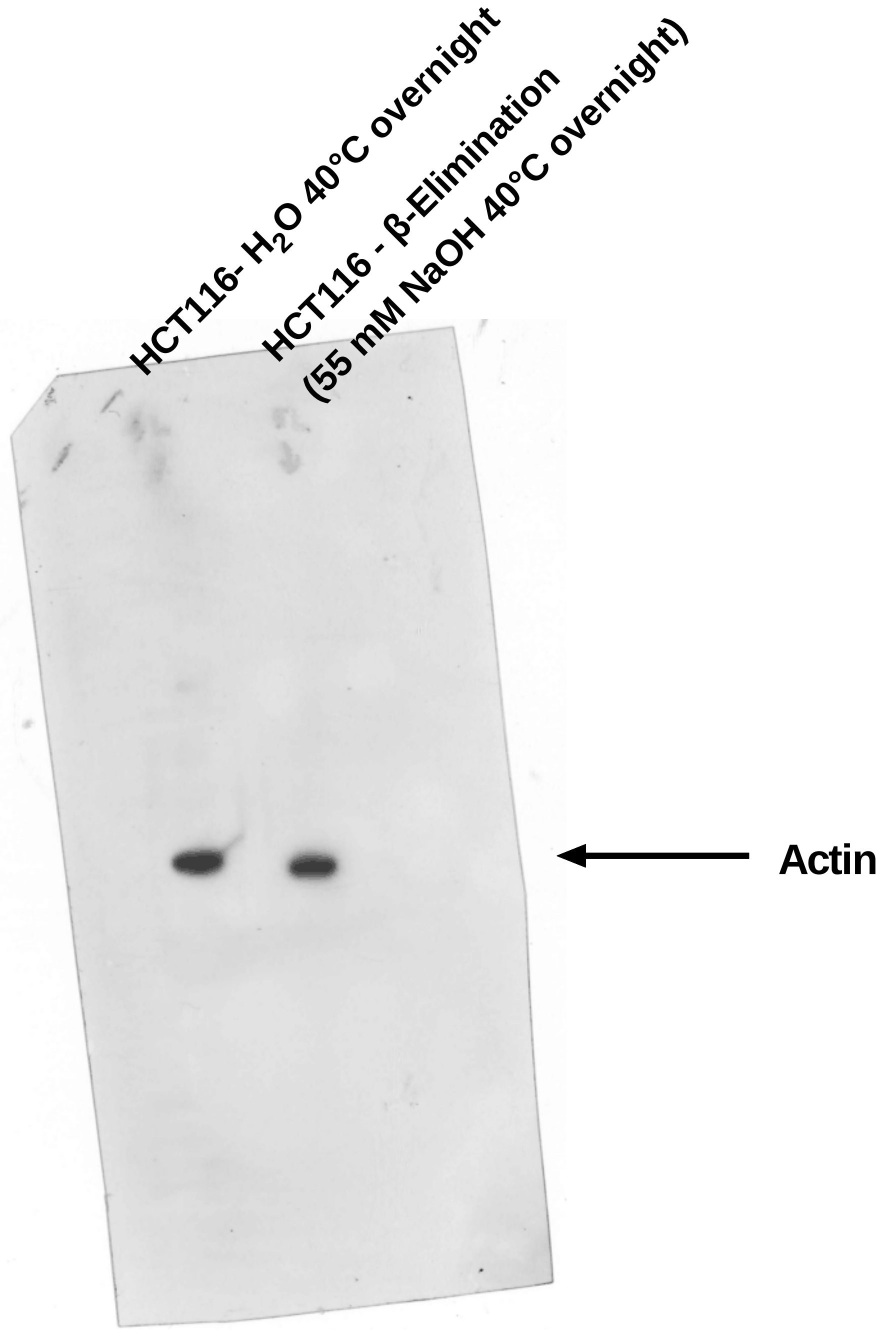

Shown in S2 Fig Lane 5-6 Panel Actin

This image was captured by using Ricoh MP2555 Scanner
