# Supplementary figures and images for: The correlation between cellular O-GlcNAcylation and sensitivity to O-GlcNAc inhibitor in colorectal cancer cells
Source: PLoS One. 2024 Oct 16;19(10):e0312173. doi: 10.1371/journal.pone.0312173 (PMC11482669; doi:10.1371/journal.pone.0312173)

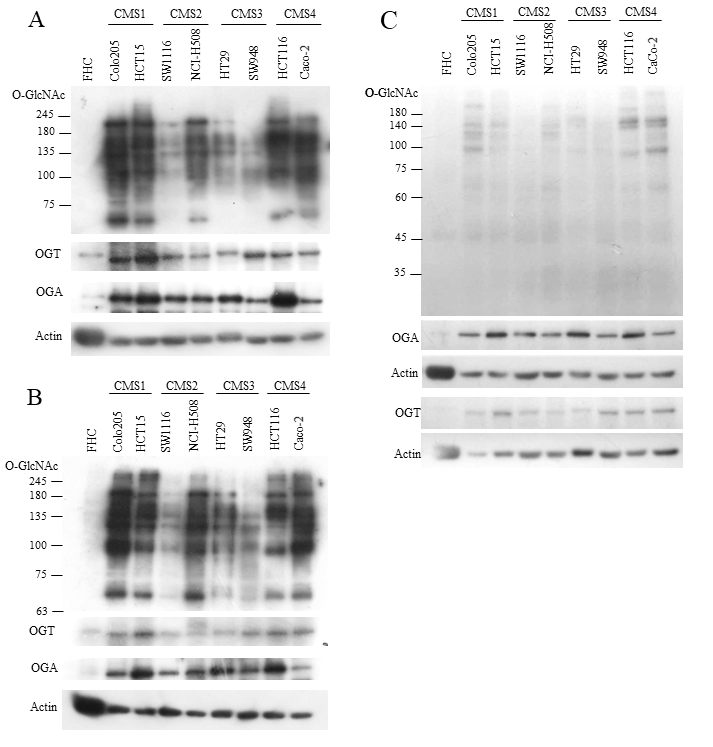

Supplement: S1 Fig — FHC cells were used as a non-cancerous cell control. (TIF) [file pone.0312173.s002.TIF]

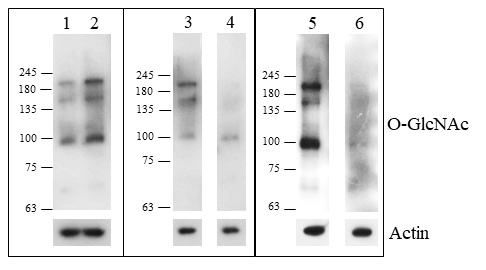

Supplement: S2 Fig — Western blot analysis of HCT116 cell lysates under various conditions: Lane 1, 3 and 5, untreated HCT116 cell lysate controls; lane 2, HCT116 treated with 1 mM DTT for 4h; lane 4, 100 mM GlcNAc blocking; and lane 6, on-blot β-elimination. Note that lane 1 and 2 were on the same blot, while lane 3–6 were duplicated blots subjected to different processing, as described in materials and methods. (TIF) [file pone.0312173.s003.TIF]

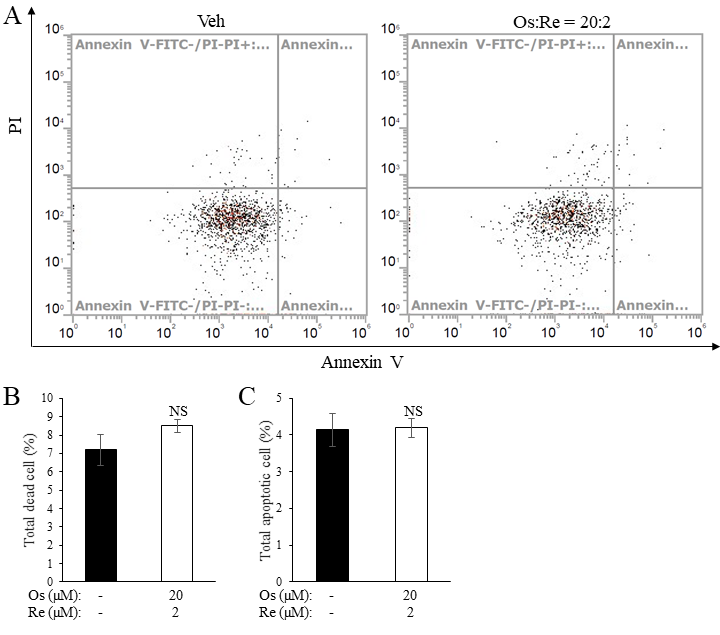

Supplement: S3 Fig — (A) Cellular apoptosis/necrosis determined by Annexin-V/PI double staining and flow-cytometry analysis. The bar graph depicted (B) total dead cells and (C) total apoptotic cells. Data presented as mean ± SD, n = 3. NS, p > 0.05. (TIF) [file pone.0312173.s004.TIF]

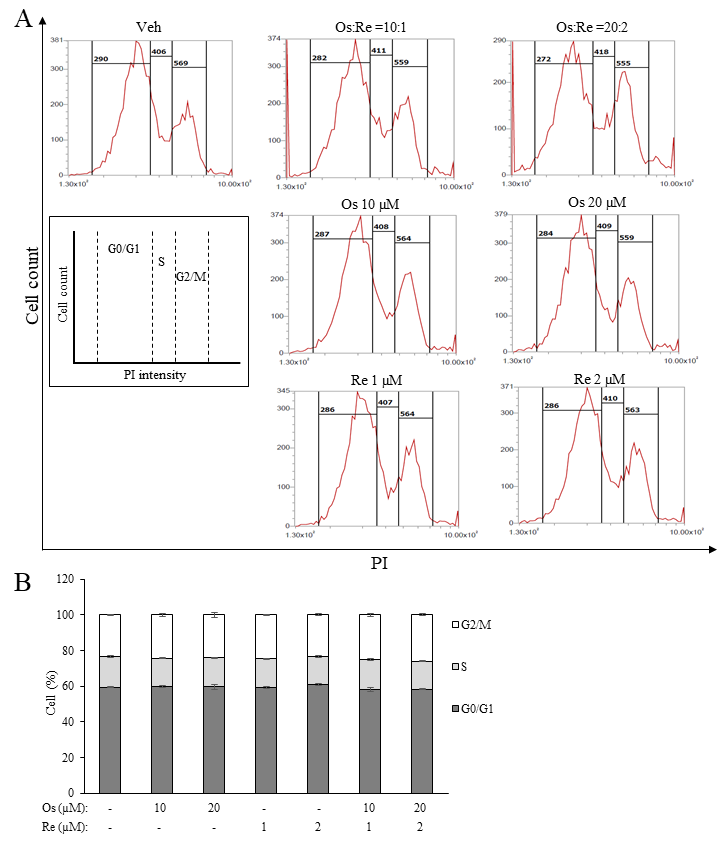

Supplement: S4 Fig — (A) Representative histogram plot of cells stained with PI analyzed by flow-cytometry. (B) Quantitation of cells in G0/G1, S and G2/M phases of the cell cycle based on PI staining status. Data presented as mean ± SD (n = 3). (TIF) [file pone.0312173.s005.TIF]
